# GNA14 stimulation of KLF7 promotes malignant growth of endometrial cancer through upregulation of HAS2

**DOI:** 10.1186/s12885-021-08202-y

**Published:** 2021-04-23

**Authors:** Jing Wang, Fei Teng, Hongxia Chai, Caixia Zhang, Xiaolei Liang, Yongxiu Yang

**Affiliations:** Department of obstetrics and gynecology, the First Hospital of Lanzhou University, Key Laboratory for Gynecologic Oncology Gansu Province, NO.1 Donggang West Road, Chengguan District, Lanzhou, Gansu 730000 P. R. China

**Keywords:** Endometrial cancer, GNA14, KLF7, HAS2, Cancer development

## Abstract

**Background:**

Endometrial cancer (UCEC) is one of the most common gynecological malignancies. We previously found that overexpression of G protein α subunit 14 (GNA14) promoted UCEC growth. Krüppel-like factor 7 (KLF7) acts as an oncogene in various cancer types, whereas the connection between GNA14 and KLF7 in UCEC is unclear. We herein explored the involvement of GNA14/KLF7 in UCEC development.

**Methods:**

Clinical relevance of GNA14, KLF7 and HAS2 in UCEC was analyzed from TCGA and by immunohistochemical staining. Knockdown and overexpression of indicated genes were conducted by transfecting the cells with siRNAs and lentivirus, respectively. mRNA and protein expression was detected by qRT-PCR and Western blot. CCK8, colony formation, cell cycle, apoptosis, transwell and wound healing were performed to check cell biology function in vitro. Tumor growth in nude mice was conducted to check in vivo function. RNA sequencing was used to determine dys-regulated genes.

**Results:**

We demonstrated that GNA14 stimulated the expression of KLF7 in UCEC cells. There was a positive correlation between GNA14 and KLF7 in normal and UCEC tissues. In vitro, KLF7 promoted cell proliferation, colony formation, cell cycle progression, and migration of UCEC cells. Apoptosis was inhibited by KLF7. Xenografted tumorigenesis of UCEC cells was suppressed by KLF7 knockdown. Furthermore, RNA sequencing results showed that KLF7 regulated the expression of a large amount of genes, among which hyaluronan synthase 2 (HAS2) was downregulated in KLF7 knockdown cells. Based on TCGA database and immunoblotting assays, KLF7 positively regulated HAS2 in UCEC cells and tissues. Lastly, knockdown of HAS2 reversed the oncogenic role of KLF7 on UCEC cell proliferation, migration, and xenografted tumor development.

**Conclusion:**

Taken together, we reveal that GNA14/KLF7/HAS2 signaling cascade exerts tumor promoting function during UCEC development.

**Supplementary Information:**

The online version contains supplementary material available at 10.1186/s12885-021-08202-y.

## Background

Endometrial cancer (UCEC) is one of the commonest malignant tumors in gynecology. During the past decades, the incidence and mortality of UCEC are rising worldwide [1]. Due to the different biological and histopathological characteristics, UCEC can be divided into two subtypes, namely type I and type II endometrial cancer [2, 3]. Development of UCEC is driven by various molecular changes, such as inactivation of tumor suppressors or activation of oncogenes. Phosphatase and tensin homolog (PTEN) represents the most frequent inactivated tumor suppressor and phosphatidylinositol-4,5-bisphosphate 3-kinase catalytic subunit alpha (PI3KCA) is the commonest mutated oncogene in UCEC. Although most of UCEC patients harboring PI3KCA response to PI3K/AKT/mTOR inhibitors treatment [4], some of them relapse and the treatment efficacy is far from satisfactory. Thus, identifying novel UCEC driven molecules may help us develop effective drugs to treat this malignant disease.

G protein α subunit 14 (GNA14), one of the G protein α subunits, plays an important role in signaling transduction, such as activation of phospholipase C, protein kinase C and MAPK pathway [5, 6]. Recent studies have shown that GNA14 may play a pivotal role in cancer development. Upregulation of GNA14 participates in the oncogenic function of tumor necrosis factor (TNF-α)/ TNF receptor superfamily member 1A (TNFR1) in gastric tumorigenesis [7]. GNA14 is frequently mutated in anastomosing haemangiomas [8]. Previously, we showed that GNA14 was upregulated in UCEC tissues and knockdown of GNA14 suppressed the proliferation and growth of UCEC cells [9]. GNA14 downregulation also induced UCEC cell apoptosis. However, the downstream molecular events of GNA14 need further investigations in UCEC development.

The Krüppel-like factors (KLFs) family is a type of transcription factor containing a conserved zinc finger domain, which regulates gene transcription activity by binding to the promoter sequence of the target genes [10, 11]. In humans, KLFs consist of 17 members, comprising of KLF1-KLF17. Based on their transcription activity, KLF7 regulates the expression of various genes and participates in cancer development. For example, KLF7 high expression predicts unfavorable prognosis of lung cancer patients. Knockdown of KLF7 inhibits the migration and invasion of lung cancer cells [12]. KLF7 is also highly expressed in squamous carcinoma. Elevated KLF7 contributes to the progression of squamous carcinoma [13]. These results suggest that KLF7 is likely an oncogene in different cancers. Nevertheless, the significance of KLF7 and its correlation with GNA14 remain to be determined in UCEC.

In this study, we analyzed the correlation between GNA14 and KLF7 in UCEC based on TCGA database and immunohistochemical staining. Then, we explored the role of KLF7 in UCEC by loss-of-function and gain-of-function experiments. Furthermore, we used RNA sequencing to investigate the downstream targets of KLF7 in UCEC. We demonstrated that GNA14/KLF7/HAS2 signaling cascade promotes UCEC progression.

## Methods

### The Cancer genome atlas database

Correlation between GNA14 and KLF7 was analyzed in normal samples and UCEC samples from The Cancer Genome Atlas (TCGA, http://cancergenome.nih.gov) database.

### Cell culture

Human UCEC cells KLE, Hec-1-A and Hec-1-B were obtained from the American Type Culture collection (ATCC, Manassas, VA, USA). All the cells were maintained in RPMI 1640 (Invitrogen, Carlsbad, CA, USA), which was supplied with 10% fetal bovine serum (FBS, Gibco, California, USA) and 1% antibiotics solution (Corning, New York, USA), at 37 °C with 5% CO_2_.

### Immunohistochemical (IHC) staining of GNA14, KLF7 and HAS2 in UCEC microarray

IHC staining of GNA14, KLF7 and HAS2 was performed in the same UCEC tissue microarray, according to the protocols as described previously [9]. Antibody against GNA14 was from Abnova (Taiwan, H00009630-M06, 1:100). KLF7 antibody was from Abcam (ab197690, Cambridge, United Kingdom, 1:100). HAS2 antibody was from Invitrogen (PA5–25593, 1:100).

### GNA14, KLF7 and HAS2 interference

siRNAs were used to interfere GNA14, KLF7 and HAS2 in UCEC cells. siRNAs (60 nM) against negative control, GNA14, KLF7 and HAS2 were purchased from GenePharma (Shanghai, China) and were transfected into KLE, Hec-1-A and Hec-1-B cells by RNAiMAX (Invitrogen). 48 h later, the cells were subjected to immunoblotting analysis of knockdown efficiency and functional experiments. For in vivo experiments, lentivirus-mediated KLF7 knockdown was performed to establish stable cell lines. siRNA sequences were as follow: siGNA14–1, 5′-CTACAGATACAGACAATAT-3′; siGNA14–2, 5′-CCAGTATCAGTTTGGTTTA-3′; siKLF7–1: 5′-CCGGCUACUUCUCAGCUUU-3′; siKLF7–2; 5′-GGUGAGGACUUGGACUGUU-3′, and siHAS2; 5′-CCAGUAUCAGUUUGGUUUA-3′. shKLF7 sequence was 5′-CCGGCUACUUCUCAGCUUU-3′.

### GNA14 and KLF7 overexpression

Lentivirus was used to overexpress GNA14 and KLF7 in UCEC cells. The coding sequence of GNA14 or KLF7 was cloned into pCDH vectors. Lentivirus was packaged in 293FT cells and were concentrated using PEG6000. The virus was harvested to infect UCEC cells and the overexpression efficiency was detected by immunoblotting. Then the cells were subjected to functional experiments.

### Immunoblotting

Total proteins were extracted from cells using RIPA buffer (Beyotime, Shanghai, China), containing protease inhibitor cocktail and phosphatase inhibitor cocktail (Roche, Basel, Switzerland). Protein concentration was detected by BCA kit (Beyotime). Subsequently, equal amount of total proteins was separated on 10–15% SDS-PAGE gels and transferred onto PDVF membranes. After incubating with 5% skim milk, primary, and secondary antibodies, the protein abundance was measured on a chemiluminescence detector. Primary antibody against GNA14 was from Abnova (Taiwan, H00009630-M06, 1:1000). HAS2 antibody was from Invitrogen (PA5–25593, 1:1000). KLF7 (sc-398,576, 1:800) and GAPDH (sc-47,724, 1:4000) antibody, and all the secondary antibodies were from SantaCruz (CA, USA, 1:8000).

### Quantitative real time PCR (qRT-PCR)

Total RNA was extracted from UCEC cells using Trizol regent (Invitrogen), following the manufacturer’s instructions. RNA was reversely transcribed using M-MLV reverse transcriptase (Promega, WI, USA). The abundance of cDNA was detected by SYBR master mixture (TransGen, Beijing, China). Primer sequences were as follow: HAS2 forward, 5′-TCCTGGATCTCATTCCTCAGC-3′, and reverse, 5′-TGCACTGAACACACCCAAAATA-3′; KLF7 forward, 5′-AGACATGCCTTGAATTGGAACG-3′, and reverse, 5′-GGGGTCTAAGCGACGGAAG-3′; GAPDH forward, 5′-TGACTTCAACAGCGACACCCA − 3′, and reverse, 5′-CACCCTGTTGCTGTAGCCAAA-3′.

### Cell proliferation

Cell proliferation was detected by CCK8 assay (40203ES60, YEASEN, Shanghai, China). A total of 2000 UCEC cells were in triplicate seeded into 96-well plates, which contained 100 ul culture medium in each well. At indicated time, 10 ul of CCK8 regent was added into each well and the plates were shocked for 30 s, followed by incubation at 37 °C for 3 h. Then OD value at 450 nm was measured on the microplate. Cell viability was normalized to the OD450 value of day 1 (OD450 value of the cells 8 h after seeding).

### Colony growth

UCEC cells were seeded into 6-well plates at equal density. 8–10 days later, colonies were formed and washed by PBS for three times. Then, they were fixed by methyl alcohol and stained by crystal violet. Lastly, the colonies were washed by clear water and dried at room temperature.

### Cell cycle

Cell cycle was detected by PI staining (40301ES50, YEASEN, Shanghai, China) and was analyzed on flow cytometry. Indicated cells were seeded in triplicate in 6-well plates. The cells were harvested and washed by PBS for two times. Then the cells were stained by PI and cell cycle was analyzed on flow cytometry.

### Apoptosis

Apoptosis was detected by PI/Annexin V staining (40302ES20, YEASEN, Shanghai, China) and was analyzed on flow cytometry. UCEC cells were seeded in triplicate in 6-well plates. The cells were trypsinized by EDTA-free trypsin and washed by PBS for two times. Then the cells were stained by PI/Annexin V and apoptosis was analyzed on flow cytometry.

### RNA sequencing

Total RNA in triplicate was extracted from UCEC cells using Trizol regent (Invitrogen), following the manufacturer’s instructions. The RNA was sent for RNA sequencing analysis by allwegene (Beijing, China). Differentially expressed genes were identified following the criterion: fold change> 1.5 and *p* < 0.05.

### In vivo tumorigenesis

6-week-old immunodeficient nude mice (female, BALB/c) were obtained from Charles River (Beijing, China). Equal number of shCtrl and shKLF7 Hec-1-B cells (1*10^7^), Ctrl, KLF7 and KLF7 + shHAS2 Hec-1-B cells (5*10^6^) were subcutaneously implanted into the mice, which were randomly divided into 5 mice per group. Tumors were formed and the mice were euthanatized 45 days after implantation. Mice were sacrificed by using carbon dioxide euthanasia method according to the protocols. Then tumors were collected for photographing and tumor weighting. All animal experiments were compliant with ethic regulations and approved by the First Hospital of Lanzhou University. The experiments were carried out according to Institutional Animal Care and Use Committee guidelines of the First Hospital of Lanzhou University.

### Transwell

Migration was assessed by transwell assay. Equal amount of siCtrl, siKLF7–1 and siKLF7–2 Hec-1-B cells in 200 ul FBS-free culture medium were seeded onto the upper layer of transwell chamber. The lower chamber contained 500 ul 10% FBS culture medium. 24 h later, the cells on the upper layer were removed and the cells on the lower layer were fixed by methyl alcohol and stained by crystal violet. Migrated cells were photographed under the microscope.

### Wound healing

Migration was assessed by wound healing assay. The cells were seeded into 6-well plates. After reaching 90% confluence, 200 ul pipettes were used to create wounds with similar breadth in each well. Cell supernatants were removed and fresh culture medium was added into each well. Cells were photographed at 0 h and 48 h later under microscope. Migration rate = (breadth of 0H- breadth of 48H)/width of 0H.

### Statistical analysis

Statistical difference was analyzed by GraphPad prism software. The experiments were carried out for three times independently. The results were presented as mean ± SEM. Student’s t test was applied to compare the difference between two groups and one-way ANOVA analysis followed by a Tukey’s post hoc test was used to analyze the difference among multiple groups. Statistical significance was considered when *p* < 0.05.

## Results

### GNA14 expression is correlated with KLF7 expression in UCEC

**Fig. 1:**
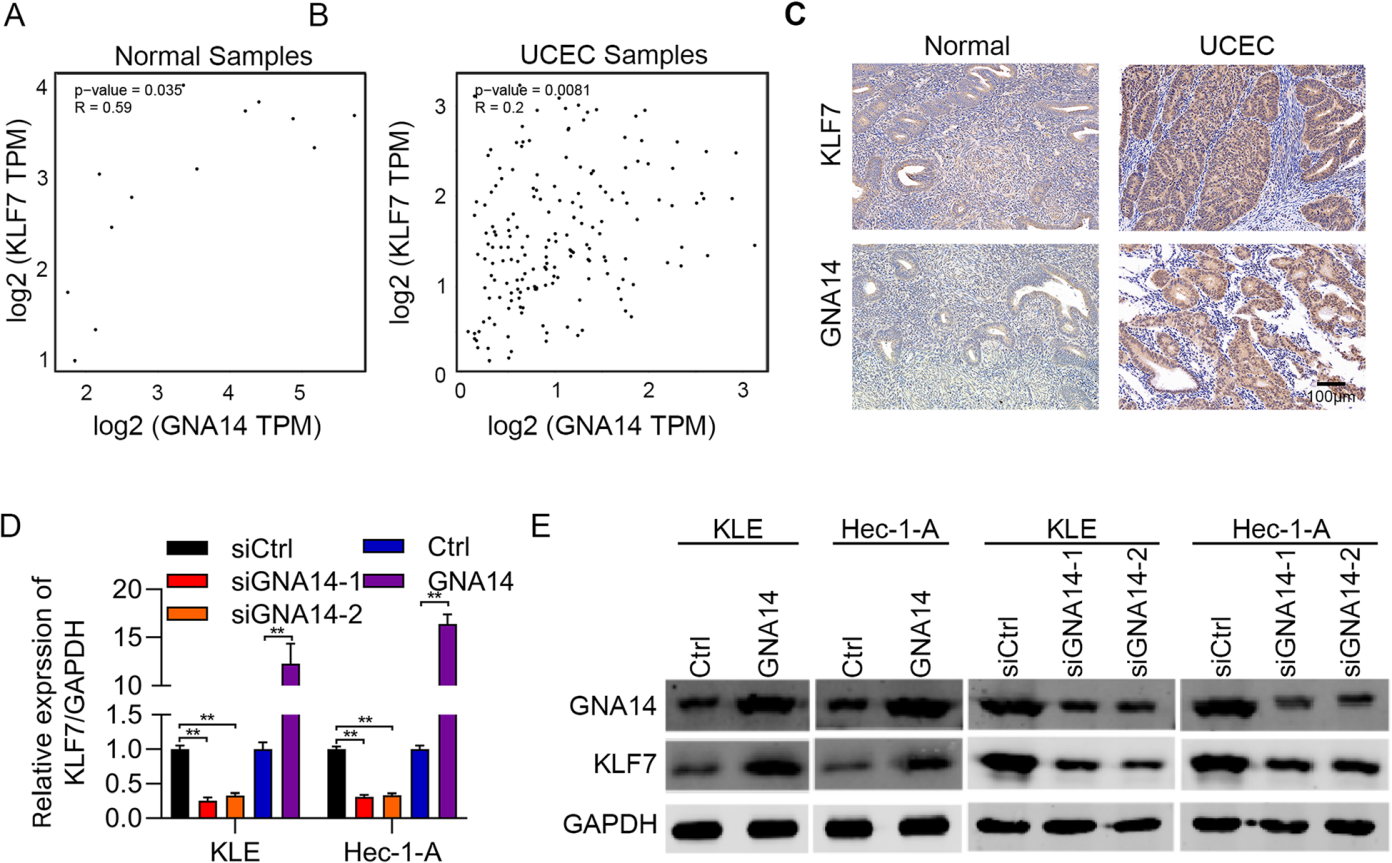
GNA14 upregulates KLF7 in UCEC. **a** and **b** The correlation between GNA14 expression and KLF7 expression was analyzed in normal samples (A, *R* = 0.59, *p* = 0.035) and UCEC samples (B, *R* = 0.2, *p* = 0.0081) from TCGA database. **c** IHC staining of GNA14 and KLF7 in UCEC (*n* = 20) and normal (*n* = 15) samples. Left, representative images of IHC staining. Right, quantification results of GNA14 and KLF7 in cancer and normal tissues. Spearman correlation between KLF7 and GNA14 was analyzed in cancer and normal samples based on IHC staining. **d** and **e** qRT-PCR (**d**) and immunoblotting (**e**) analysis of GAN14 and KLF7 in GAN14 knockdown and overexpressed Hec-1-A and KLE cells. GAPDH acts as the internal control. **p < 0.01

We previously found that GNA14 was an oncogene in UCEC. To explore the downstream mechanisms of GNA14 in UCEC, we firstly analyzed the positively correlated genes of GNA14 from TCGA database. We observed that there was a positive relationship between GNA14 and KLF7 mRNA abundance in both normal and UCEC tissues (Fig. 1a and b). To strengthen this result, we performed immunohistochemical staining of GNA14 and KLF7 in UCEC and normal samples. We showed that both GNA14 and KLF7 were overexpressed in UCEC tissues (Fig. 1c). In addition, GNA14 was positively correlated with KLF7 in these samples (Tables 1-2). We therefore investigated whether GNA14 regulated KLF7 in UCEC cells by knocking down and overexpressing GNA14. qRT-PCR and immunoblotting results showed that GNA14 knockdown reduced the mRNA and protein expression of KLF7, whereas GNA14 overexpression promoted the mRNA and protein expression KLF7 in UCEC cells (Fig. 1d and e). Taken together, GNA14 positively regulated KLF7 in UCEC cells and tissues.

**Table 1:** The expression of KLF7, GNA14 and HAS2 in normal tissue and endometrial cancer by IHC

	Tumor Tissue	Normal tissue	χ^2^	*p* Value
KLF7				
High expression	2	35	8.81	< 0.01
Low expression	8	15		
GNA14				
High expression	3	37	6.67	< 0.01
Low expression	7	13		
HAS2				
High expression	4	38	5.14	0.02
Low expression	6	12		
Total	10	50

**Table 2:** Spearman correlation analysis of expression among KLF7, GNA14, and HAS2 in 50 endometrial cancer tissues by IHC

	KLF7		GNA14	
	*r*_*s*_	*P* value	*r*_*s*_	*P* value
GNA14	0.573	< 0.001		
HAS2	0.583	< 0.001	0.632	< 0.001

r, Spearman correlation

### KLF7 promotes the proliferation and migration of UCEC cells

**Fig. 2:**
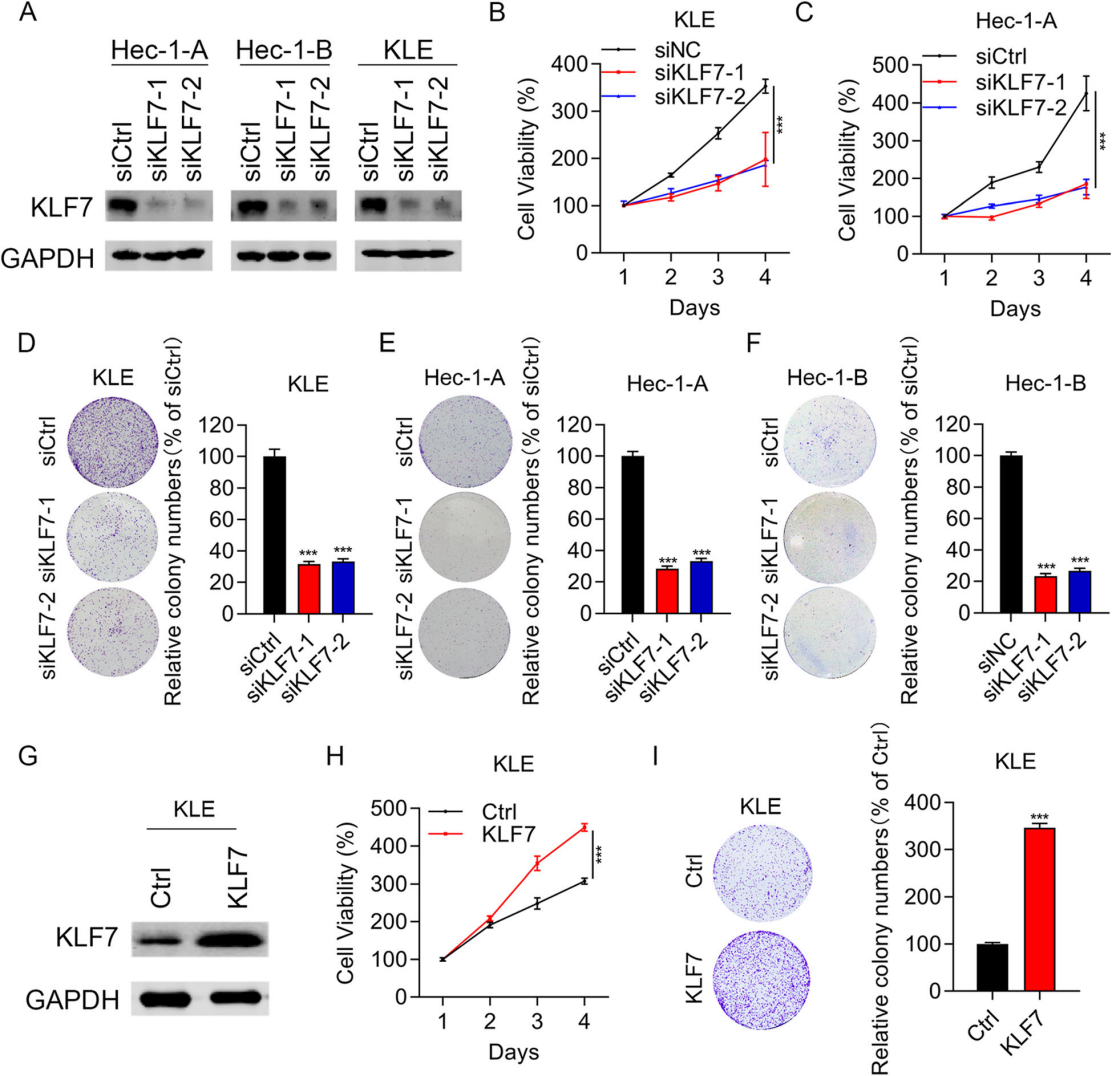
KLF7 promotes UCEC cell proliferation. **a** Immunoblotting analysis of KLF7 in siCtrl, siKLF7–1 and siKLF7–2 UCEC cells. **b** and **c** CCK8 analysis of cell proliferation in siCtrl, siKLF7–1 and siKLF7–2 KLE and Hec-1-A cells. **d-f** Colony formation was assessed in siCtrl, siKLF7–1 and siKLF7–2 KLE, Hec-1-A, and Hec-1-B cells. **g** Immunoblotting analysis of KLF7 in Ctrl and KLF7 overexpressed KLE cells. **h** CCK8 analysis of cell proliferation in Ctrl and KLF7 overexpressed KLE cells. **i** Colony formation was assessed in Ctrl and KLF7 overexpressed KLE cells. ***p < 0.001. Results are mean ± SEM (*n* = 3)

Since GAN14 is an oncogene and is positively correlated with KLF7 in UCEC, we next intend to explore the function of KLF7 in UCEC. KLF7 was knocked down using siRNAs in UCEC cells and the cells were subjected to cell proliferation and migration experiments. Immunoblotting results showed that KLF7 was obviously silenced in siKLF7–1 and siKLF7–2 cells (Fig. 2a). Interference of KLF7 significantly suppressed the proliferation of KLE and Hec-1-A cells (Fig. 2b and c). In addition, KLF7 knockdown also led to reduced colony growth in KLE, Hec-1-A and Hec-1-B cells (Fig. 2d-f). To address whether KLF7 is sufficient to induce the phenotypes, we overexpressed KLF7 in KLE cells (Fig. 2g). We found that KLF7 ectopic expression enhanced the proliferation and colony formation ability of KLE cells (Fig. 2h and i). These results suggest that KLF7 acts as an oncogene in UCEC.

### KLF7 promotes cell cycle progression and suppresses apoptosis of UCEC cells

**Fig. 3:**
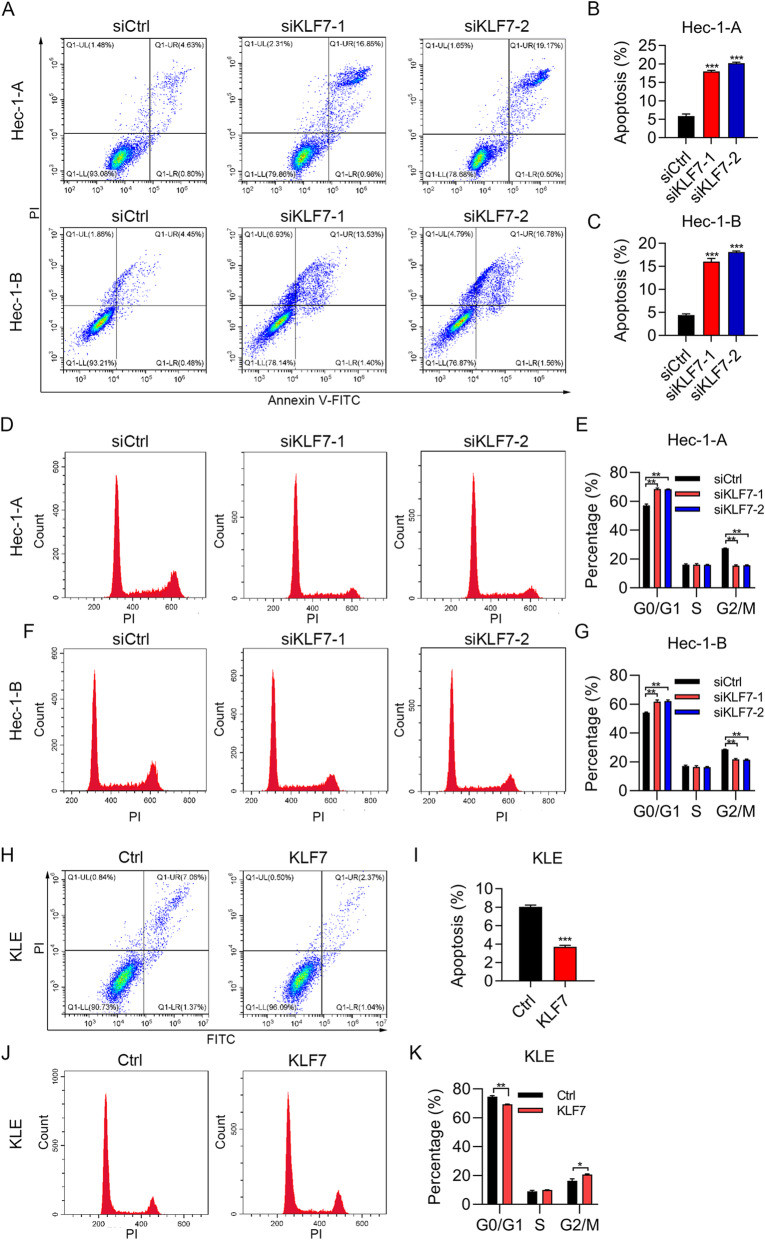
KLF7 regulates cell cycle and apoptosis in UCEC cells. **a-c** Apoptosis was examined by PI/Annexin V staining in siCtrl, siKLF7–1 and siKLF7–2 Hec-1-A and Hec-1-B cells. **a**, apoptosis results. **b** and **c**, quantitative results. **d-g** Cell cycle was examined by PI staining in siCtrl, siKLF7–1 and siKLF7–2 Hec-1-A and Hec-1-B cells. **d** and **f**, cell cycle results. **e** and **g**, quantitative results. **h** and **i** Apoptosis was examined by PI/Annexin V staining in Ctrl and KLF7 overexpressed KLE cells. **h**, apoptosis results. **i**, quantitative results. **j** and **k** Cell cycle was examined by PI staining in Ctrl and KLF7 overexpressed KLE cells. **j**, cell cycle results. **k**, quantitative results. *p < 0.05. **p < 0.01. ***p < 0.001. Results are mean ± SEM (*n =* 3)

To study the effect of KLF7 on cell cycle progression and apoptosis, we subjected the cells to PI staining and PI/Annexin V staining. We found that KLF7 knockdown led to enhanced apoptosis in Hec-1-A and Hec-1-B cells (Fig. 3a-c). To define the type of cell death, we next treated the cells with caspase inhibitor Z-VAD-FMK and necroptotic inhibitor necrostatin-1, and then detected cell viability. We showed that Z-VAD-FMK, but not necrostatin-1, reversed the cell death caused by KLF7 knockdown. These results confirmed that cell death promoted by KLF7 knockdown was apoptosis but not necrosis (Supplementary Fig. 1A and B). PI staining results showed that KLF7 knockdown led to cell cycle arrest at G0/G1 phase in Hec-1-A and Hec-1-B cells (Fig. 3d-g). By contrast, KLF7 ectopic expression resulted in decreased apoptosis and reduced cell number of G0/G1 phase in Hec-1-A and Hec-1-B cells (Fig. 3h-k). Taken together, KLF7 promotes cell proliferation by regulating apoptosis and cell cycle progression.

### KLF7 promotes UCEC cell migration

**Fig. 4:**
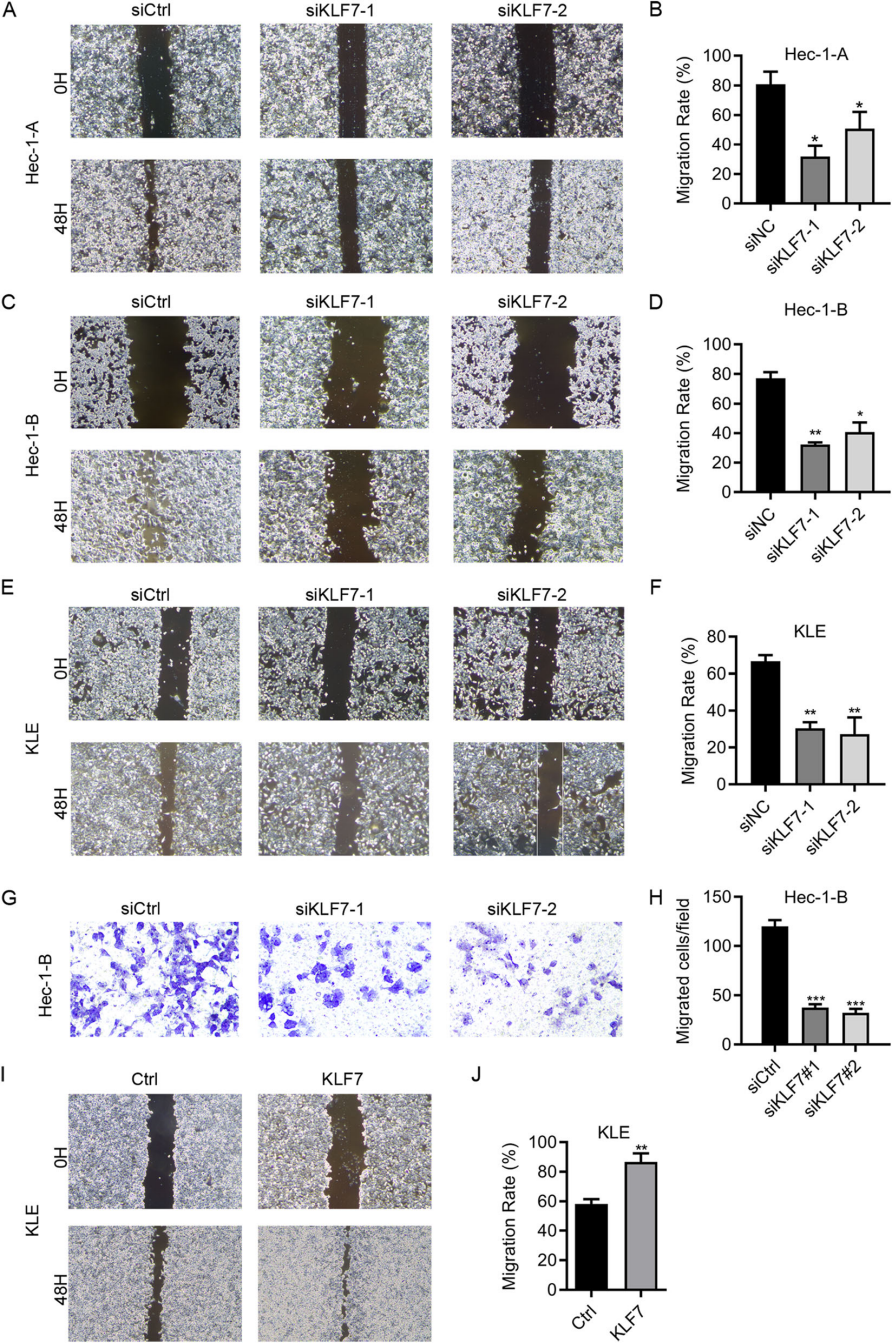
KLF7 promotes UCEC cell migration. **a-f** Migration was determined by wound healing assay in siCtrl, siKLF7–1 and siKLF7–2 Hec-1-A (**a** and **b**), Hec-1-B (**c** and **d**), and KLE (**e** and **f**) cells. **g** and **h** Transwell detection of migration in siCtrl, siKLF7–1 and siKLF7–2 Hec-1-B cells. **i** and **j** Migration was determined by wound healing assay in Ctrl and KLF7 overexpressed KLE cells. *p < 0.05. **p < 0.01. ***p < 0.001. Results are mean ± SEM (*n =* 3)

Next, we assessed the role of KLF7 on UCEC cell migration by using wound healing and transwell assay. Would healing results showed that knockdown of KLF7 significantly suppressed the migration of Hec-1-A, Hec-1-B and KLE cells (Fig. 4a-f). Moreover, the migration capacity of Hec-1-B cells was reduced after KLF7 knockdown as shown by transwell assay (Fig. 4g and h). Furthermore, KLF7 overexpression enhanced the migration of KLE cells (Fig. 4i and j). Above results indicate that KLF7 expression is critical for UCEC cell migration. Thus, KLF7 contributes to UCEC cell migration.

### KLF7 knockdown results in dys-regulation of numerous genes

**Fig. 5:**
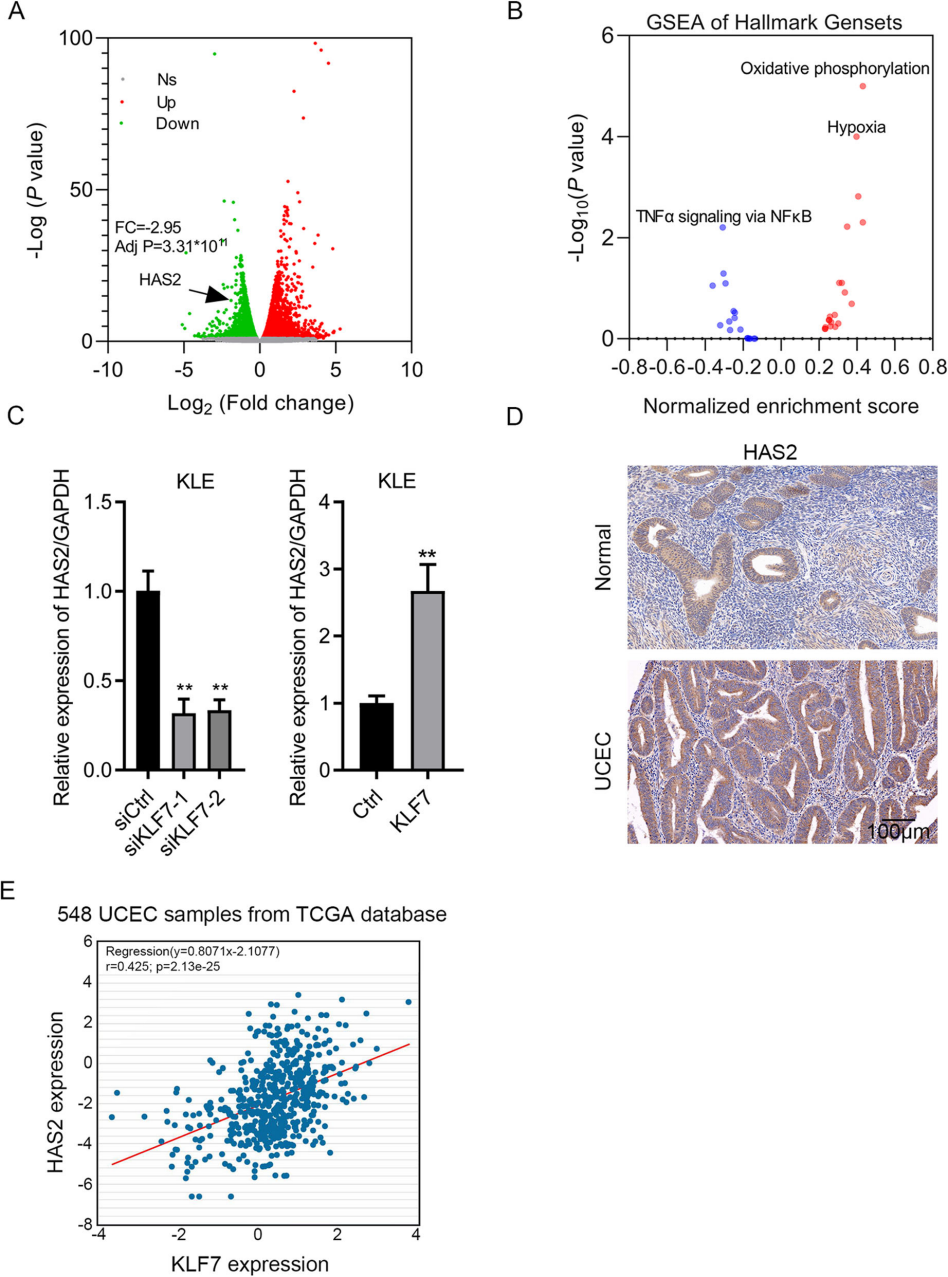
Molecule profiling in UCEC cells after KLF7 knockdown. **a** siCtrl and siKLF7 UCEC cells were subjected to RNA sequencing. Differentially expressed genes were identified following the criterion: fold change> 1.5 and *p <* 0.05. **a** Upregulated genes were shown as red. Downregulated genes were shown as blue. Gray represented unchanged genes. **b** GESA analysis of signaling pathway after KLF7 knockdown. **c** qRT-PCR detection of HAS2 in KLE cells after KLF7 knockdown and overexpression. **d** Correlation between KLF7 expression and HAS2 expression was analyzed in UCEC tissues base on TCGA database. **p < 0.01. Results are mean ± SEM (*n =* 3)

To profile the downstream effectors of KLF7 in UCEC cells, we subjected siCtrl and siKLF7 UCEC cells to RNA sequencing analysis. Differentially expressed genes were identified following the criterion: fold change> 1.5 and *p* < 0.05. We showed that a total of 2464 genes were upregulated and a total of 2377 genes were downregulated after KLF7 knockdown (Fig. 5a and Table S1). GSEA analysis showed that TNFα signaling via NF-kB was suppressed, while oxidative phosphorylation and hypoxia were activated after KLF7 knockdown (Fig. 5b). We also found that HAS2 was downregulated in siKLF7 cells based on RNA sequencing data. qRT-PCR showed that HAS2 expression was decreased and increased in KLF7 knockdown and overexpressed cells, respectively (Fig. 5c). Then, we checked the expression of HAS2 in UCEC tissues by IHC staining and observed that HAS2 was overexpressed in UCEC tissues and was positively correlated with the expression of KLF7 (Fig. 5d and Tables 1-2). TCGA database analysis showed that there was a positive correlation between KLF7 expression and HAS2 expression in UCEC tissues (Fig. 5e).

Because KLF7 was positively regulated by GNA14 (Fig. 1e and Table 2), we then examined whether GNA14 regulated the expression of HAS2. We showed that GNA14 overexpression upregulated HAS2, while GNA14 knockdown suppressed the expression of HAS2 in HEC-1-A and KLE cells (Supplementary Fig. 2). Taken together, GNA14 upregulation of KLF7 promotes the expression of HAS2 in UCEC cells and tissues.

### HAS2 acts as an oncogene in UCEC

**Fig. 6:**
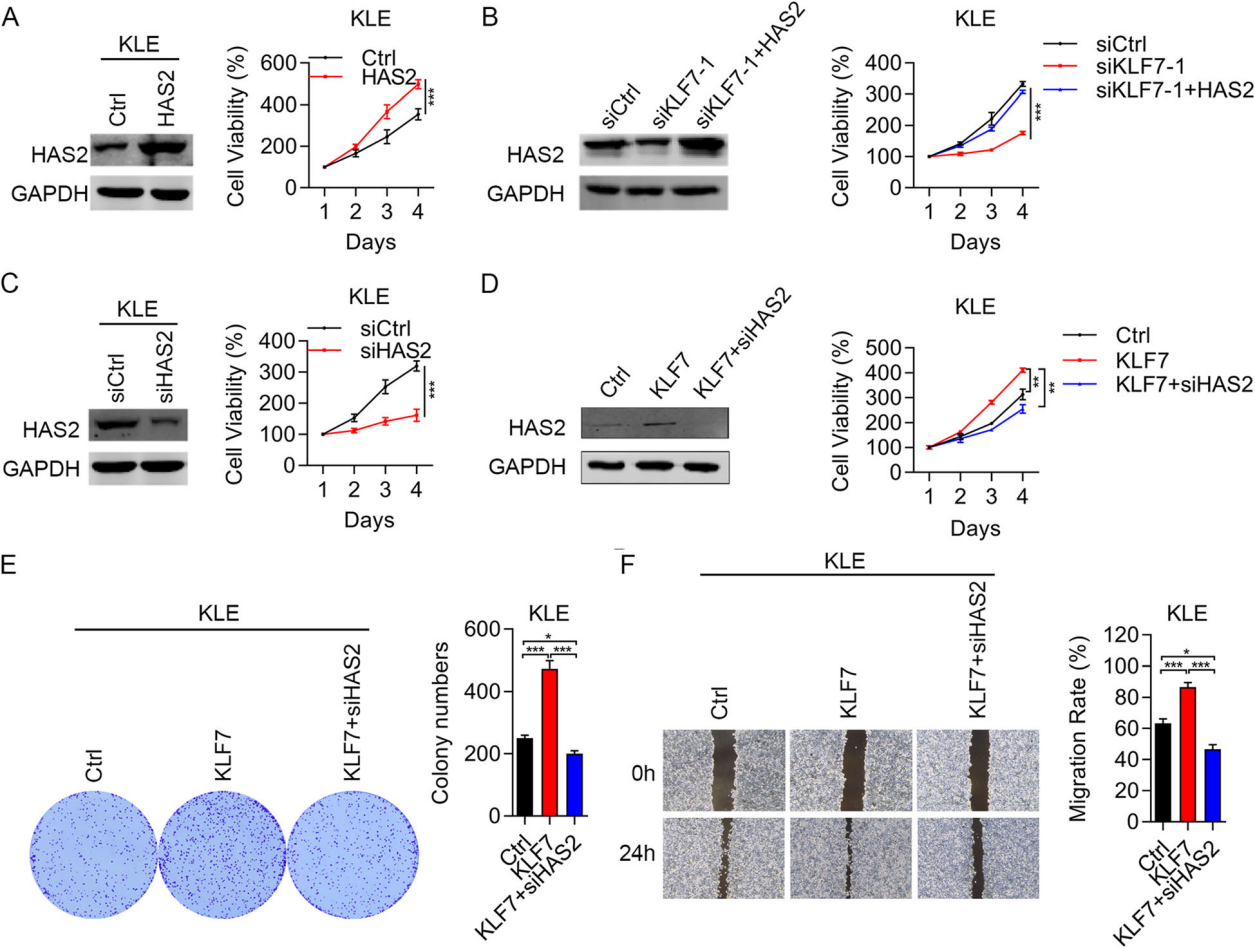
KLF7 upregulation of HAS2 promotes UCEC cell proliferation and migration. **a-d** Immunoblotting analysis of HAS2 and cell viability were checked in Ctrl and HAS2 overexpressed KLE cells (**a**), in siCtrl, siKLF7 and siKLF7 + HAS2 KLE cells (**b**), in siCtrl and siHAS2 KLE cells (**c**), and in Ctrl, KLF7 and KLF7 + siHAS2 KLE cells (**d**). **e** CCK8 analysis of proliferation in Ctrl, KLF7 and KLF7 + siHAS2 KLE cells. **f** Colony formation was assessed in Ctrl, KLF7 and KLF7 + siHAS2 KLE cells. **g** Migration was assessed by wound healing in Ctrl, KLF7 and KLF7 + siHAS2 KLE cells. *p < 0.05. **p < 0.01. ***p < 0.001. Results are mean ± SEM (*n =* 3)

RNA sequencing and qRT-PCR results showed that KLF7 upregulated HAS2, but whether HAS2 contributes to the oncogenic function of KLF7 in UCEC should be determined. To address this question, we firstly overexpressed HAS2 in KLE cells. We showed that HAS2 overexpression stimulated the proliferation ability of KLE cells (Fig. 6a). Then, we overexpressed HAS2 in KLF7 silenced cells and found that HAS2 upregulation restored the suppressed cell proliferation caused by KLF7 knockdown (Fig. 6b). By contrast, we knocked down HAS2 in KLE cells and observed that HAS2 downregulation inhibited KLE cell growth (Fig. 6c). Next, we silenced HAS2 in KLF7 overexpressed KLE cells. Immunoblotting results showed that KLF7 overexpression upregulated HAS2 in KLE cells, and HAS2 knockdown efficiently downregulated HAS2 in KLE cells (Fig. 6d). CCK8 and colony formation results demonstrated that HAS2 silencing suppressed the cell proliferation and colony formation in KLF7 overexpressed KLE cells (Fig. 6d and e). Furthermore, HAS2 knockdown inhibited the migration in KLF7 overexpressed KLE cells (Fig. 6f). Therefore, HAS2 upregulation by KLF7 promotes UCEC cell proliferation and migration.

### KLF7 upregulation of HAS2 contributes to UCEC cell tumorigenesis in nude mice

**Fig. 7:**
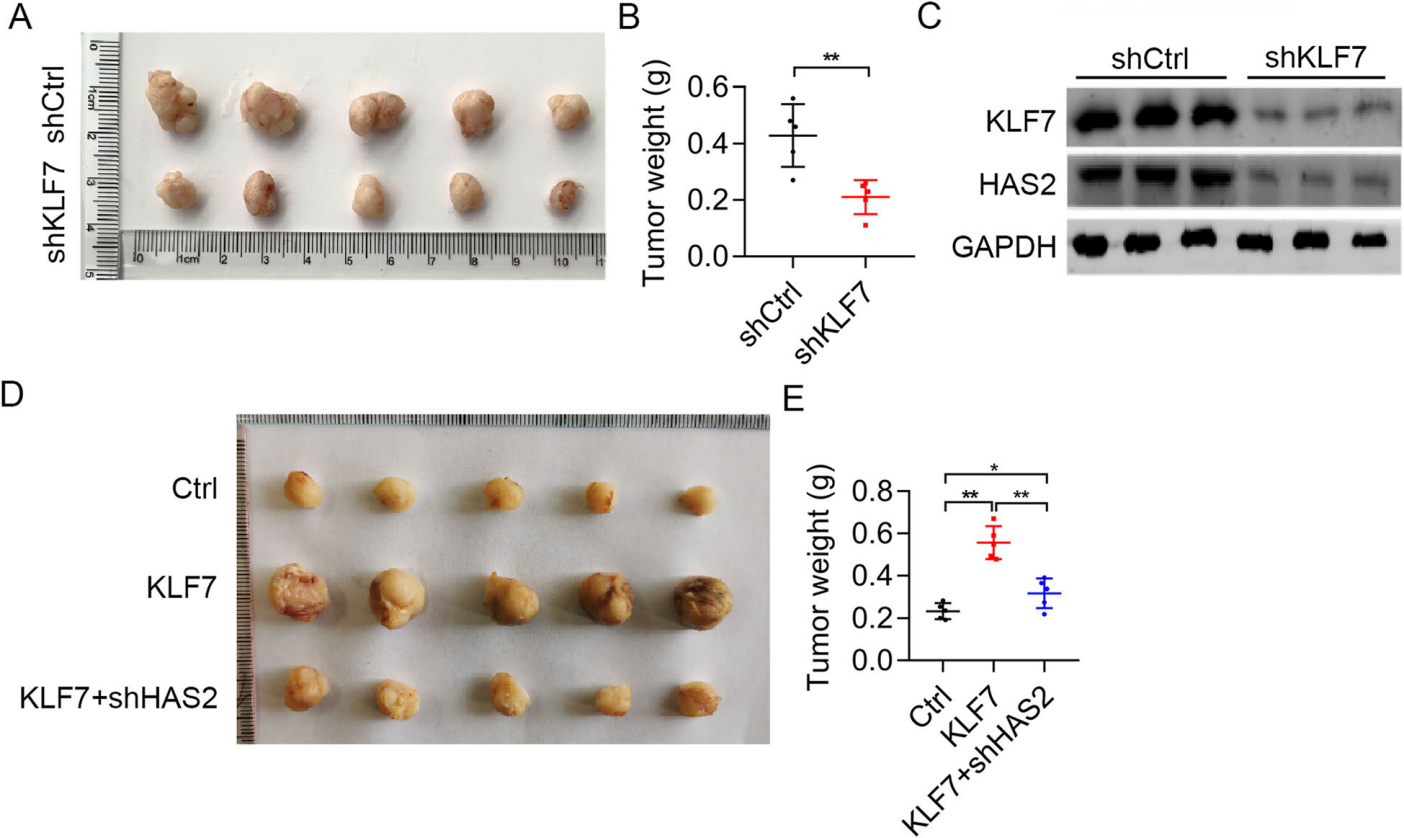
KLF7 promotes tumorigenesis of UCEC cells through upregulation of HAS2. Equal number of shCtrl and shKLF7 Hec-1-B cells were subcutaneously implanted into female nude mice. Tumors were photographed after sacrifice. **a** Macroscopic images of tumors derived from shCtrl and shKLF7 Hec-1-B cells in nude mice. **b** Tumor weight was measured. **c** Immunoblotting analysis of KLF7 and HAS2 in shCtrl and shKLF7 tumor tissues. Equal number of Ctrl, KLF7, KLF7 + shHAS2 Hec-1-B cells were subcutaneously implanted into female nude mice. Tumors were photographed after sacrifice. **d** Macroscopic images of tumors derived from Ctrl, KLF7, KLF7 + shHAS2 Hec-1-B cells in nude mice. **e** Tumor weight was measured. *p < 0.05. **p < 0.01

Next, we explored the in vivo role of KLF7 in UCEC. Hec-1-B cells expressing shCtrl and shKLF7 lentivirus were subcutaneously injected into the immunodeficient nude mice (6-week-old, male BALB/c) to develop xenografted tumor models. Tumor growth was evaluated and tumor weight was assessed after sacrifice. We found that KLF7 knockdown suppressed the growth of Hec-1-B cells in nude mice (Fig. 7a and b). Moreover, the tumors were subjected to immunoblotting analysis of KLF7 and HAS2. The results showed that both KLF7 and HAS2 were downregualted in shKLF7 tumor tissues as compared shCtrl tissues (Fig. 7c).

Lastly, we explored whether KLF7/HAS2 cascade promoted xenograted tumor development by implanting equal number of Hec-1-B cells expressing Ctrl, KLF7 overexpressing, and KLF7 overexpressing plus shHAS2 lentiviruis onto female nude mice. We showed that KLF7 overexpressing accelerated xenograted tumor development of Hec-1-B cells, which could be reversed by HAS2 knockdown (Fig. 7d and e). Therefore, KLF7 upregulation of HAS2 contributes to UCEC tumor growth.

## Discussion

Endometrial cancer (UCEC) is one of the most common gynecological malignancies, accounting for 20–30% of all female reproductive system cancers. In recent years, scientists have made many efforts to analyze the mechanism of the occurrence and development of endometrial cancer. The progression of UCEC is closely related to the abnormalities of KRAS, PTEN and β-catenin [14–16]. Although the above genes play an important role in the occurrence and development of endometrial cancer, it is still difficult to fully analyze the tumorigenesis and development of all endometrial cancer patients. The pathogenic mechanism of endometrial cancer still needs to be further explored.

We previously found that GNA14 was highly expressed in UCEC patients. Knockdown of GNA14 blocked cell growth and induced apoptosis in UCEC cells [9]. These results suggest that GNA14 is likely a promising drug target in UCEC. However, the downstream molecular mechanisms should be investigated. In this study, we found that KLF7 was positively regulated by GNA14 in UCEC cells. Interestingly, there was a positive correlation between GNA14 and KLF7 in UCEC patients. Thus, KLF7 might be the downstream effector of GNA14 in UCEC. As a transcription factor, KLF7 participates in the development of various cancers. For instance, KLF7 is negatively regulated by P53 pathway and upregulation of KLF7 promotes the development of pancreatic ductal adenocarcinoma by regulating golgi complex integrity [17]. KLF7 is also overexpressed in gastric cancer samples and it encourages the migration and invasion of gastric cancer cells [18]. Furthermore, KLF7 can also be targeted by microRNA, such as miR-185 and miR-193a, in lung cancer [19, 20]. Overall, KLF7 functions as on oncogene in different cancers and its upregulation can be regulated by various factors. Based on the results as demonstrated by other studies, we predicted that GNA14 upregulation of KLF7 might partly contribute to UCEC development. As expected, loss-of-function and gain-of-function experiments demonstrated that KLF7 encouraged cell cycle progression, proliferation, colony growth, migration, and tumorigenesis of UCEC cells. However, when GNA14 knockdown led to G2/M arrest, downregulation of KLF7 resulted in G0/G1 arrest in cell cycle progression. This implied that KLF7 was not the only downstream effector of GNA14. There might be some genes related to cell cycle progression that could be regulated by GNA14 bypass KLF7. These questions will be investigated in the future. Furthermore, apoptosis was inhibited by KLF7 in UCEC cells. Therefore, we concluded that KLF7 contributed to the oncogenic role of GNA14 in UCEC.

Because KLF7 is a transcription factor, there may be a downstream effector of KLF7 in UCEC. Based on our RNA sequencing and validation data, we showed that KLF7 positively regulated the expression of hyaluronan synthase 2 (HAS2), which is a critical enzyme controlling the biosynthesis of hyaluronan. A study showed that HAS2 regulation of hyaluronan synthesis was important for liver fibrosis development [21]. Recently, a large numbers of studies have reported the function and significance of HAS2 in cancer development. HAS2 increases breast cancer cell invasion through inhibiting tissue metalloproteinase inhibitor 1 (TIMP-1) [22]. Silencing of HAS2 promotes the radiosensitivity of cancer cells [23]. Overexpression of HAS2 is correlated with malignant function of breast cancer cells [24]. Although hyaluronan synthase and its product hyaluronan are likely related to the progression of UCEC [25–27], there are no direct evidences which demonstrate the oncogenic role of HAS in UCEC. Furthermore, the upstream regulators of HAS2 in UCEC are underdetermined. We here found that KLF7 served as a positive regulator of HAS2 in UCEC cells. TCGA database showed a positive correlation between KLF7 and HAS2. We also demonstrated that HAS2 knockdown reversed the phenotypes observed in KLF7 overexpressed UCEC cells. Taken together, KLF7 upregulation of HAS2 promotes UCEC proliferation.

There are some limitations in this previous study. 1. We only focused on KLF7 as a GNA14 substrate in UCEC. The functions of other KLFs in UCEC were not involved in this study. 2. We did not check whether KLF7 knockdown or overexpression regulates the cellular functions of normal endometrial cells. In the future, we will investigate whether other family members of KLFs act as the downstream effectors of GNA14 and also the role of other KLFs in UCEC development.

## Conclusions

In summary, we provided the evidence that GNA14/KLF7/HAS2 signaling cascade promoted the development of UCEC. This signaling cascade was also a promising diagnostic candidate for UCEC. Blocking this cascade may benefit UCEC patients.

## Supplementary Information

**Additional file 1: Supplementary Fig. 1**. KLF7 regulates apoptosis but not necrosis. (A and B) siCtrl, siKLF7–1 and siKLF7–2 Hec-1-A (A) and KLE (B) cells were incubated with vehicle, Z-VAD-FMK and Necrostatin-1 for 24 h. Then cell viability was detected by CCK8 assay. *p < 0.05. **p < 0.01. Results are mean ± SEM (*n* = 3).

**Additional file 2 Supplementary Fig. 2**. GNA14 promotes the expression of HAS2 in UCEC cells. Immunoblotting analysis of HAS2 in siCtrl, siGNA14–1, siGNA14–2, and in Ctrl and GNA14 overexpressed KLE and Hec-1-A cells. The expression of HAS2 was adjusted to GAPDH.

**Additional file 3 **: **Supplementary Fig. 3**. Full-length Western blot images.

**Additional file 4: Supplementary Tab. 1.** Gene expression after KLF7 knockdown in KLE cells.

## Data Availability

The datasets used and/or analysed during the current study are available from the corresponding author on reasonable request.

## Abbreviations

UCEC: Endometrial cancer
GNA14: G protein α subunit 14
KLFs: Krüppel-like factors
HAS2: Hyaluronan synthase 2
ATCC: American Type Culture collection
FBS: Fetal bovine serum
qRT-PCR: Quantitative Real Time PCR
TIMP-1: Inhibiting tissue metalloproteinase inhibitor 1
PTEN: Phosphatase and tensin homolog
PIK3CA: Phosphatidylinositol-4,5-bisphosphate 3-kinase catalytic subunit alpha
TNF: Tumor necrosis factor
TNFR1: TNF receptor superfamily member 1A
