# Supplementary material for: GNA14 stimulation of KLF7 promotes malignant growth of endometrial cancer through upregulation of HAS2
Source: BMC Cancer. 2021 Apr 23;21:456. doi: 10.1186/s12885-021-08202-y (PMC8066949; doi:10.1186/s12885-021-08202-y)
Supplement: Supplementary file 1 — Additional file 1: Supplementary Fig. 1. KLF7 regulates apoptosis but not necrosis. (A and B) siCtrl, siKLF7–1 and siKLF7–2 Hec-1-A (A) and KLE (B) cells were incubated with vehicle, Z-VAD-FMK and Necrostatin-1 for 24 h. Then cell viability was detected by CCK8 assay. *p < 0.05. **p < 0.01. Results are mean ± SEM (n = 3). [file 12885_2021_8202_MOESM1_ESM.docx]

**Supplementary Fig. 1. KLF7 regulates apoptosis but not necrosis.**


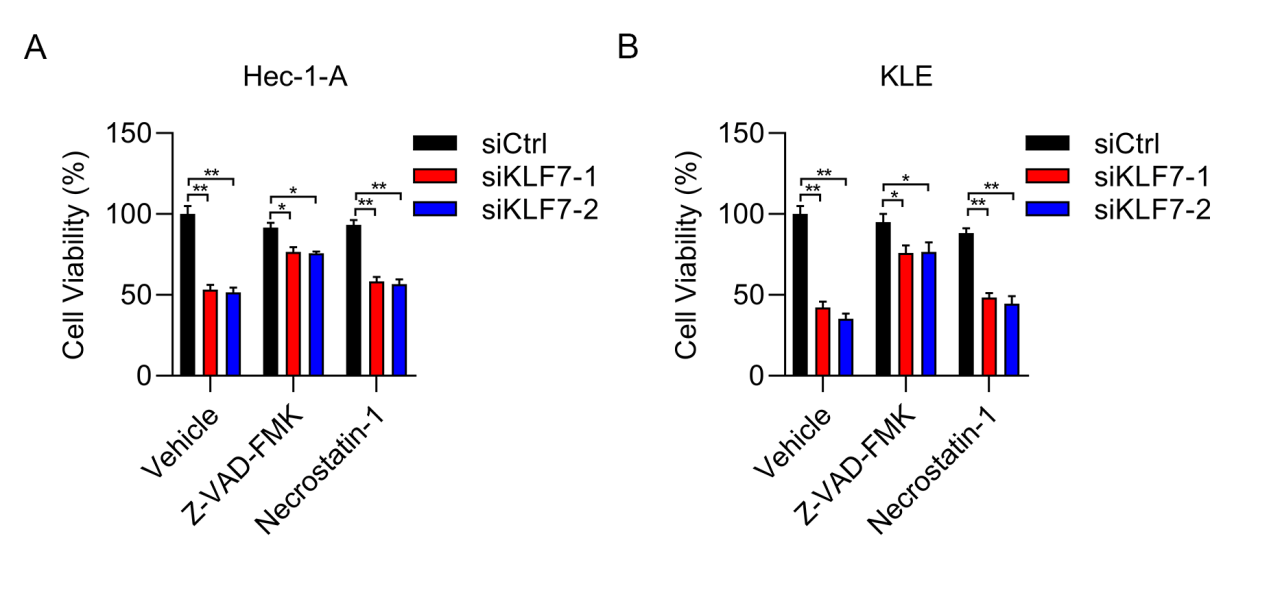


(A and B) siCtrl, siKLF7-1 and siKLF7-2 Hec-1-A (A) and KLE (B) cells were incubated with vehicle, Z-VAD-FMK and Necrostatin-1 for 24 hours. Then cell viability was detected by CCK8 assay. *p<0.05. **p<0.01. Results are mean ± SEM (n=3).
