# Supplementary material for: GNA14 stimulation of KLF7 promotes malignant growth of endometrial cancer through upregulation of HAS2
Source: BMC Cancer. 2021 Apr 23;21:456. doi: 10.1186/s12885-021-08202-y (PMC8066949; doi:10.1186/s12885-021-08202-y)
Supplement: Supplementary file 2 — Additional file 2 Supplementary Fig. 2. GNA14 promotes the expression of HAS2 in UCEC cells. Immunoblotting analysis of HAS2 in siCtrl, siGNA14–1, siGNA14–2, and in Ctrl and GNA14 overexpressed KLE and Hec-1-A cells. The expression of HAS2 was adjusted to GAPDH. [file 12885_2021_8202_MOESM2_ESM.docx]

**Supplementary Fig. 2. GNA14 promotes the expression of HAS2 in UCEC cells.**


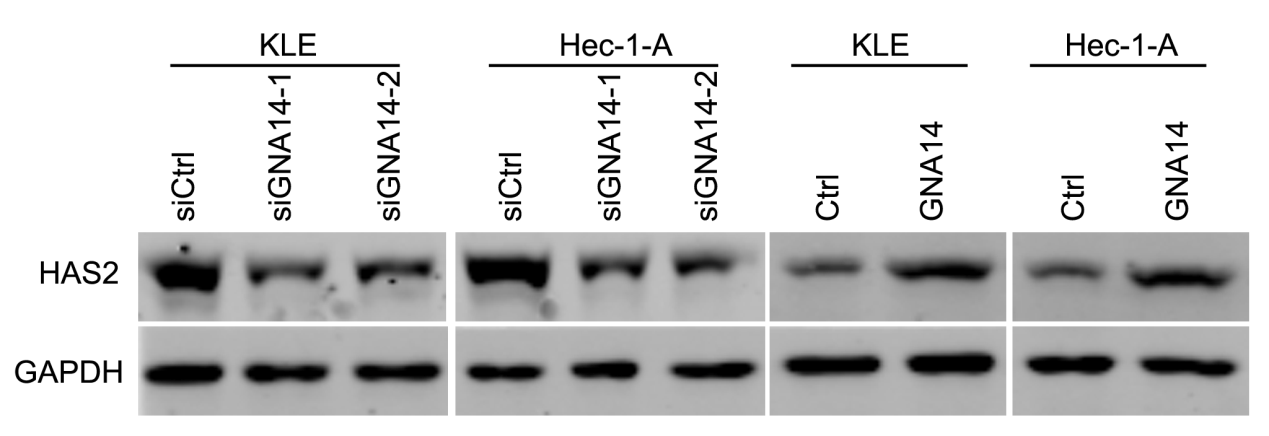


Immunoblotting analysis of HAS2 in siCtrl, siGNA14-1, siGNA14-2, and in Ctrl and GNA14 overexpressed KLE and Hec-1-A cells. The expression of HAS2 was adjusted to GAPDH.
