# Supplementary figures and images for: GNA14 stimulation of KLF7 promotes malignant growth of endometrial cancer through upregulation of HAS2
Source: BMC Cancer. 2021 Apr 23;21:456. doi: 10.1186/s12885-021-08202-y (PMC8066949; doi:10.1186/s12885-021-08202-y)

**Supplementary Fig. 3. Full-length Western blot images.**

**
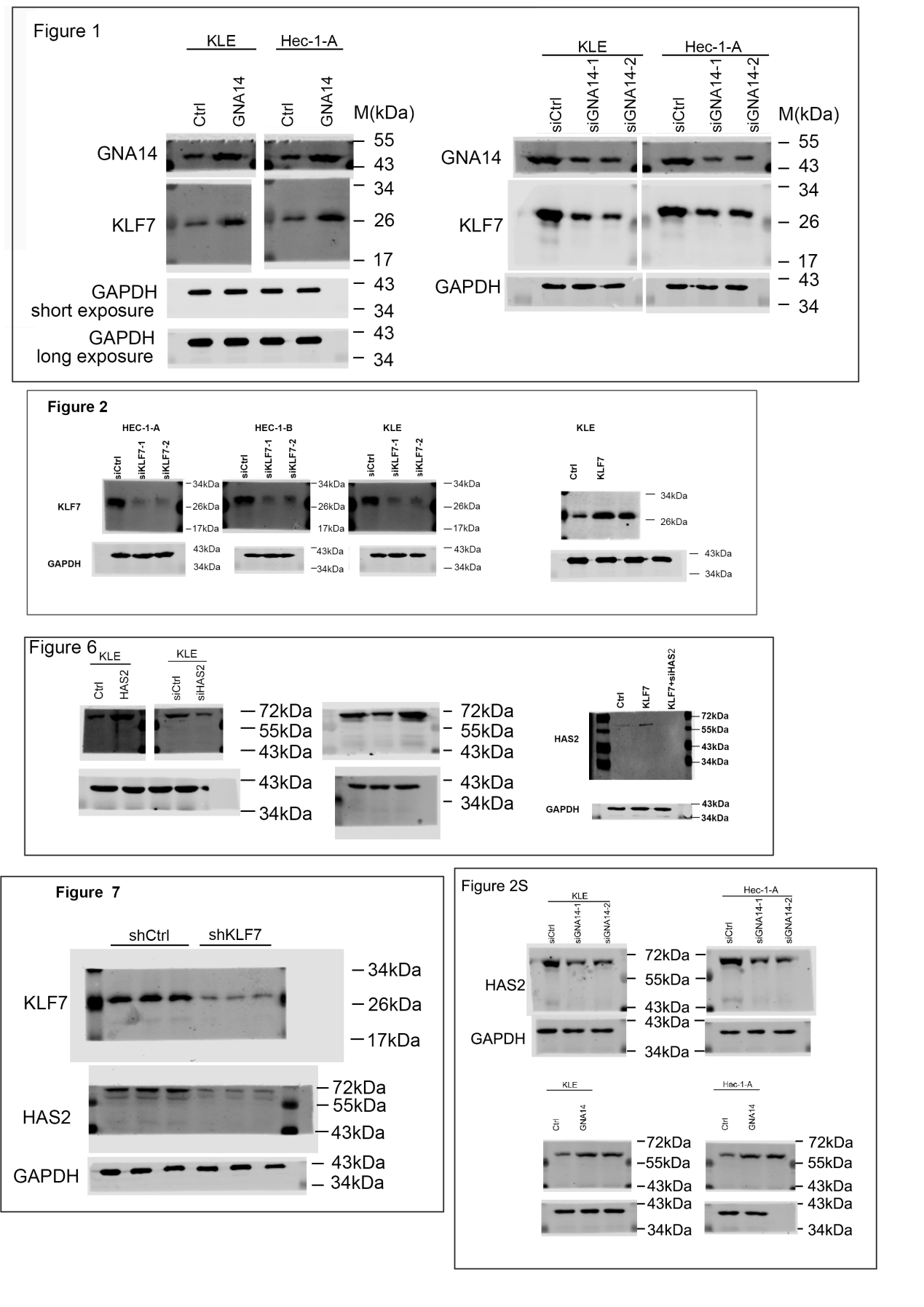
**

Supplement: Supplementary file 3 — Additional file 3 : Supplementary Fig. 3. Full-length Western blot images. [file 12885_2021_8202_MOESM3_ESM.docx]
